# Development and application of a quadruplex *TaqMan* real-time fluorescence quantitative PCR assay for four porcine digestive pathogens

**DOI:** 10.3389/fcimb.2024.1468783

**Published:** 2024-11-26

**Authors:** Haojie Wang, Yue Sun, Jianxing Chen, Wei Wang, Haibo Yu, Caixia Gao, Tongqing An, Yue Wang, Hongyan Chen, Liangquan Zhu, Zhimin Jin, Changqing Yu, Changyou Xia, He Zhang

**Affiliations:** ^1^ State Key Laboratory for Animal Disease Control and Prevention, Harbin Veterinary Research Institute, Chinese Academy of Agricultural Sciences, Harbin, China; ^2^ College of Life Sciences and Technology, Mudanjiang Normal University, Mudanjiang, China; ^3^ China Institute of Veterinary Drug Control, Beijing, China; ^4^ School of Advanced Agricultural Sciences, Yibin Vocational and Technical College, Yibin, China

**Keywords:** bacterial gastrointestinal diseases, Salmonella, Escherichia coli, Lawsonella intracellularis, Brachyspira hyodysenteriae, Quadruplex TaqMan real-time fluorescence quantitative PCR

## Abstract

**Introduction:**

*Salmonella*, *Escherichia coli*, *Lawsonella intracellularis*, and *Brachyspira hyodysenteriae* are the primary pathogens responsible for gastrointestinal diseases in pigs, posing a significant threat to the health and productivity of pig production systems. Pathogen detection is a crucial tool for monitoring and managing these infections.

**Methods:**

We designed primers and probes targeting the *invA* gene of *Salmonella*, the 23S *rRNA* gene of *Escherichia coli*, the *aspA* gene of *Lawsonella intracellularis*, and the *nox* gene of *Brachyspira hyodysenteriae*. We developed a quadruplex TaqMan real-time quantitative PCR assay capable of simultaneously detecting these four pathogens.

**Results:**

This assay demonstrated high sensitivity, with detection limits of 100 copies/μL for the recombinant plasmid standards pEASY-23S rRNA, pEASY-aspA, and pEASY-*nox*, and 10 copies/μL for pEASY-*invA*. The standard curves exhibited excellent linearity (R^2^ values of 0.999, 0.999, 1, and 0.998, respectively) and high amplification efficiencies (93.57%, 94.84%, 85.15%, and 81.81%, respectively). The assay showed high specificity, with no cross-reactivity detected against nucleic acids from Streptococcus suis, porcine epidemic diarrhoea virus (PEDV), porcine transmissible gastroenteritis virus (TGEV), *Pasteurella multocida*, *Clostridium perfringens*, *Gracilaria parapsilosis*, porcine delta coronavirus (PDCoV), porcine group A rotavirus (GARV), and porcine teschovirus (PTV). The assay also exhibited excellent repeatability, with inter- and intra-assay coefficient of variation (CV) ranging from 0.15% to 1.12%. High concentrations of nucleic acids did not interfere with the detection of low concentrations, ensuring robust performance in complex samples. Among 263 diarrhoeic samples, the assay detected *Salmonella* in 23.95%, *Escherichia coli* in 26.24%, *Lawsonella intracellularis* in 33.84%, and *Brachyspira hyodysenteriae* in 22.43%.

**Discussion:**

This quadruplex TaqMan qPCR assay offers a rapid, sensitive, and specific tool for the simultaneous detection of *Salmonella*, *Escherichia coli*, *Lawsonella intracellularis*, and *Brachyspira hyodysenteriae* in pigs.

## 1 Introduction

With the increasing density of pig farming, porcine digestive diseases have become progressively more complex (Choi Y. et al., 2024). The misuse of antibiotics, along with the rise in drug-resistant bacteria, has made monitoring bacterial digestive diseases even more critical (Subramani et al., 2023). Notably, pathogens such as *Salmonella* (SE), *Escherichia coli* (*E. coli*), *Lawsonia intracellularis* (*L. intracellularis*, LI), and *Brachyspira hyodysenteriae* (*B. hyodysenteriae*, B.h) can induce symptoms like diarrhea, enteritis, growth retardation, and depression in pigs. These symptoms are often difficult to differentiate clinically. Moreover, these four pathogens are prone to causing mixed infections, which lead to increased morbidity and mortality, contributing to substantial economic losses in the pig industry (Chanamé Pinedo et al., 2024; Collins and Collins, 2024; Pérez-Pérez et al., 2024; Parihar et al., 2024). Additionally, SE and *E. coli* are zoonotic pathogens that can infect humans through contaminated food or water, causing gastrointestinal illnesses. This poses a significant risk to public health, as these infections can result in widespread transmission (Choi N. et al., 2024; Wang Z. et al., 2024).

Porcine salmonellosis, also known as porcine paratyphoid fever, is caused by SE, a Gram-negative, ovoid, facultatively anaerobic, non-budding, non-spore-forming, flagellated, and motile bacterium (Yoon et al., 2024). Over 2000 serotypes of Salmonella have been identified, with more than 200 found in China (Zhang et al., 2024). The serotypes primarily responsible for this disease include *Salmonella Choleraesuis*, *Salmonella typhimurium*, and *Salmonella enteritidis*, with *Salmonella Choleraesuis* and *Salmonella typhimurium* being the most significant (Ojha et al., 2024). Acute cases of the disease often present as septicemia, while chronic cases result in necrotizing inflammation of the large intestine (Flórez-Delgado et al., 2023). The disease predominantly affects piglets between 1 to 4 months of age, with cases in adult pigs being rare worldwide (Bearson, 2022). In the United States, an estimated 1.3 million SE infections and 420 associated deaths occur annually, with an economic burden of approximately $3.7 billion. Moreover, more than 50% of US hog farms test positive for SE (Bearson, 2022).

Porcine colibacillosis is an enteric disease primarily affecting piglets, caused by pathogenic *E. coli* (Siddi et al., 2024). Pathogenic *E. coli* can be classified into diarrheagenic *E. coli* and extraintestinal pathogenic *E. coli* (Li X. et al., 2024). Diarrheagenic *E. coli* is further subdivided into six categories: enteropathogenic *E. coli*, enterotoxigenic *E. coli*, enteroinvasive *E. coli*, enterohemorrhagic *E. coli*, enteroaggregative *E. coli*, and diffusely adherent *E. coli* (Thystrup et al., 2024). Extraintestinal pathogenic *E. coli* includes strains such as uropathogenic *E. coli* (Klemberg et al., 2024). This disease predominantly affects newborn piglets within their first week of life (Klemberg et al., 2024) and is clinically characterized by yellow, watery diarrhea and rapid onset of death (Wang F. et al., 2024). *E. coli* is currently the most prevalent bacterium in pig farms, with the highest isolation rates. Studies indicate that the isolation rate of porcine *E. coli* in northeastern China reaches 88% (176/200) (Cheng et al., 2021).

Porcine proliferative enteropathy (PPE) is caused by LI, a Gram-negative, weakly aerobic, curved or S-shaped, non-ciliated, non-spore-forming bacterium (Park et al., 2024). The pathogen primarily infects the ileum and colon (Salazar et al., 2023). The chronic form of the disease is the most common, characterized by diarrhea, soft brown feces, a rough coat, and reduced feed intake and weight gain (Salazar et al., 2023). A survey of 47 farms in China reported a clinical sample positivity rate of 37.3% (332/891) and a farm positivity rate of 93.6% (Wang L. et al., 2024). Although PPE has a low direct mortality rate (1%–10%), it significantly reduces growth rates, and mortality can reach 40%–50% when secondary infections occur (Barbosa et al., 2023). The presence of other enteric pathogens, such as B.h, colonic spirochetes, and SE, may exacerbate PPE when co-infections occur on the same farm (de Groot et al., 2022; Daniel et al., 2023).

Swine dysentery is a severe hemorrhagic disease of the intestinal mucosa in pigs caused by B.h (Dors et al., 2019). Clinically, the disease manifests as severe hemorrhagic dysentery in acute cases, while subacute and chronic cases are characterized by mucosal diarrhea that persists over time. B.h is a Gram-negative, serpentine, spiral-shaped, strictly anaerobic bacterium from the spirochete family (Hakimi et al., 2024). Under natural conditions, pigs of all ages and breeds are susceptible to infection, though piglets aged 2 to 3 months are most commonly affected (Scherrer and Stephan, 2021). Morbidity and mortality rates are higher in younger pigs compared to older ones. A survey conducted in six European countries reported that the prevalence of B.h in herds with a history of diarrhea ranged from 4.2% to 45.8% (Arnold et al., 2023).

In summary, the four aforementioned pathogenic bacteria exhibit high prevalence and cause significant damage in porcine gastrointestinal diseases. They are prone to mixed or secondary infections, making clinical differentiation challenging (de Groot et al., 2022; Parihar et al., 2024). Traditional microbiological and serological methods are cumbersome, time-consuming, labor-intensive, and often lack sufficient sensitivity and specificity (Song et al., 2015; Barac et al., 2024). Moreover, single conventional PCR and single fluorescent quantitative PCR methods are limited to detecting only one pathogen at a time, making them unsuitable for differential diagnosis in cases of mixed infections (Wang L. et al., 2024). To address these challenges, we targeted the *invA* gene of SE, the *23s rRNA* gene of *E. coli*, the *aspA* gene of LI, and the *nox* gene of B.h to design specific primers and probes. We developed a quadruplex TaqMan fluorescence quantitative PCR assay to simultaneously detect SE, *E. coli*, LI, and B.h, providing an efficient and convenient tool for the prevention and control of swine gastrointestinal diseases.

## 2 Materials and methods

### 2.1 Bacteria and viruses

This laboratory maintains strains of *Escherichia coli O157, Salmonella Choleraesuis, Brachyspira hyodysenteriae, Lawsonia intracellularis, Streptococcus suis (S. suis, SS)*, Porcine epidemic diarrhea virus (PEDV), Swine transmissible gastroenteritis virus (TGEV), *Pasteurella multocida (P. multocida, Pm), Clostridium welchii, Gracilaria parapsilosis (G. parapsilosis, GPS)*, Porcine delta coronavirus (PDCoV), Porcine group A rotavirus (PoRVA), and Porcine teschovirus (PTV).

### 2.2 Primer and probe design and recombinant plasmid synthesis

The sequences of the *invA* gene from SE (GenBank ID: CP053865), the *23s rRNA* gene from *E. coli* (GenBank ID: CP136755.1), the *aspA* gene from LI (GenBank ID: CP107054.1), and the *nox* gene from B.h (GenBank ID: KU215621.1) were aligned with target sequences using BLASTN in BLAST. Sequences showing high similarity or consistency with the target genes were downloaded and analyzed for specific and conserved regions using Geneious Primer and Oligo 7 software. The conserved regions were selected for the synthesis of plasmid standards: pEASY-*invA*, pEASY-23s rRNA, pEASY-*aspA*, and pEASY-*nox*. Specific primers and probes for quantitative PCR were designed, with the 5′ end labeled with FAM, VIC, Texas Red, and Cy5 fluorescent reporter dyes, and the 3′ end labeled with the corresponding MGB fluorescence quenching groups. Primers and probes demonstrating strong specificity and high sensitivity were identified through experimental screening (**Table 1**).

**Table 1:** Fluorescence quantitative PCR primers and probes.

Pathogens	Gene	Sequence(5′-3′)	Product size(bp)
*Salmonella*	*invA*	F: CATTTACGCGGGTCACGATA R:CAGATCCCCGCATTGTTGA Probe: FAM-CGGCACTAATCGCA-MGB	58
*Escherichia coli*	*23s rRNA*	F: GGCAGTCAGAGGCGATGAAG R: TTCGGAAATCGCCGGTTATA Probe: VIC- TAAGCGTCGGTAAGGTGATA -MGB	85
*Lawsonia intracellularis*	*aspA*	F:ATCCACAGCGAGGACCACTT R: CGGGTGCTTATGTTCAGCTTT Probe: Texas Red - AACTGTAACTCTTTTAAGAACAC-MGB	105
*Brachyspira hyodysenteriae*	*nox*	F:ATAGAAGCATTCAAAAACCATGGTAA R:TTTCAGCTTCATCAGTGATTTCTTTATC Probe: Cy5-TTATCTTAATGGAAGCTATGCC-MGB	103

### 2.3 Reaction system optimization

First, single-target fluorescence quantitative PCR amplification was performed, using the lowest cycle threshold (Ct value) and the highest ΔRn as indicators for optimization. The annealing temperature was optimized within a range of 58°C to 62°C, while the primer and probe concentrations were optimized between 0.1 μM and 0.5 μM. The cycle numbers were tested at 35, 40, 45, and 50. Based on the optimized conditions from the single-target fluorescence quantitative PCR, the reaction system and conditions for the quadruplex fluorescence quantitative PCR assay were established.

### 2.4 Standard curves and minimum detection limits

The recombinant plasmid standards pEASY-invA, pEASY-23s rRNA, pEASY-aspA, and pEASY-nox were diluted from 4 × 10^10^ copies/μL to 4 × 10^0^ copies/μL using a gradient of TE buffer. These dilutions were then mixed in equal volumes to achieve final concentrations ranging from 1 × 10^10^ copies/μL to 1 × 10^0^ copy/μL. The assay was subsequently performed using the optimized quadruplex fluorescence quantitative PCR assay.

### 2.5 Specificity test

DNA/RNA was extracted from SS, PEDV, TGEV, Pm, *Clostridium welchii*, GPS, PDCoV, PoRVA, and PTV as non-target genes. The recombinant plasmid standards pEASY-invA, pEASY-23s rRNA, pEASY-aspA, and pEASY-nox served as positive controls, while sterile water was used as a blank control. These samples were detected using the established quadruplex TaqMan real-time fluorescence quantitative PCR assay.

### 2.6 Interference and repeatability tests

Plasmid concentrations of 10^7^ and 10^3^ copies/µL were selected, and standard plasmid concentrations of SE, *E. coli*, LI, and B.h were randomly combined. Three parallel samples were prepared for each experiment to observe changes in threshold values at low concentrations, assessing whether high concentrations affected amplification at low concentrations. The recombinant plasmids of SE, *E. coli*, LI, and B.h were used as templates for both inter- and intra-batch experiments, and the standard deviation and coefficient of variation of the Ct values were calculated.

### 2.7 Clinical sample testing

From June 2023 to February 2024, a total of 263 samples (including anal swabs, feces, intestines, etc.) exhibiting symptoms of swine digestive tract disease were collected from various pig farms in Heilongjiang Province. Nucleic acids were extracted from these samples using commercially available kits and subsequently analyzed using the established quadruplex TaqMan real-time fluorescence quantitative PCR assay. Additionally, the extracted nucleic acids were assessed using a previously reported standard method to compare compliance rates (DB34/T2162-2014; SN/T1207-2003; SN/T3488-2013; SN/T5439.5-2022).

## 3 Results and analysis

### 3.1 Results of optimization of reaction system and reaction conditions

Following the optimization of primers, probes, annealing temperature, and cycle number for single fluorescence quantitative PCR, the optimal annealing temperature was established at 59°C. The final concentrations of the primers and probes are detailed in **Table 2**, which correspond to the lowest Ct values and the highest fluorescence signals. At cycle numbers of 20, 45, and 50, insufficient amplification of the target DNA or non-specific amplification was observed. In contrast, a cycle number of 40 yielded optimal amplification. Building on the optimization of the single fluorescence quantitative PCR reaction conditions, further adjustments were made to the concentrations of primers and probes, leading to the finalization of the quadruplex TaqMan real-time fluorescence quantitative PCR reaction system (**Table 3**) and the corresponding reaction conditions (**Table 4**).

**Table 2:** Optimization of primers and concentrations for single heavy fluorescent quantitative PCR.

Pathogens	Final concentration (μM)	
	Forward and reverse primer	TaqMan probe
*Salmonella*	0.2	0.1
*Escherichia coli*	0.3	0.3
*Lawsonia intracellularis*	0.2	0.4
*Brachyspira hyodysenteriae*	0.15	0.2

**Table 3:** The quadruplex *TaqMan* real-time fluorescence quantitative PCR reaction system.

Reagent	Volume(μL)
2 × Animal Detection U + Probe qPCR Super PreMix	12.5
invA-F(10 μM)	0.25
invA-R(10 μM)	0.25
invA-Probe(10 μM)	0.2
23s rRNA-F(10 μM)	0.3
23s rRNA-R(10 μM)	0.3
23s rRNA- Probe(10 μM)	0.25
aspA-F(10 μM)	0.25
aspA-R(10 μM)	0.25
aspA-Probe(10 μM)	0.25
nox-F(10 μM)	0.4
nox- R(10 μM)	0.4
nox- Probe(10 μM)	0.3
50 × ROX Reference Dye 2	0.5
Template DNA	2
ddH_2_O	6.6
Total	25

**Table 4:** The quadruplex *TaqMan* real-time fluorescence quantitative PCR reaction program.

Step	Time
contamination digestion 37°C	2min
Premutability 95°C	30s
Denaturation 95°C Annealing and collection of fluorescent signals 59°C	10s }40 30s

### 3.2 Standard curves

**Figure 1:**
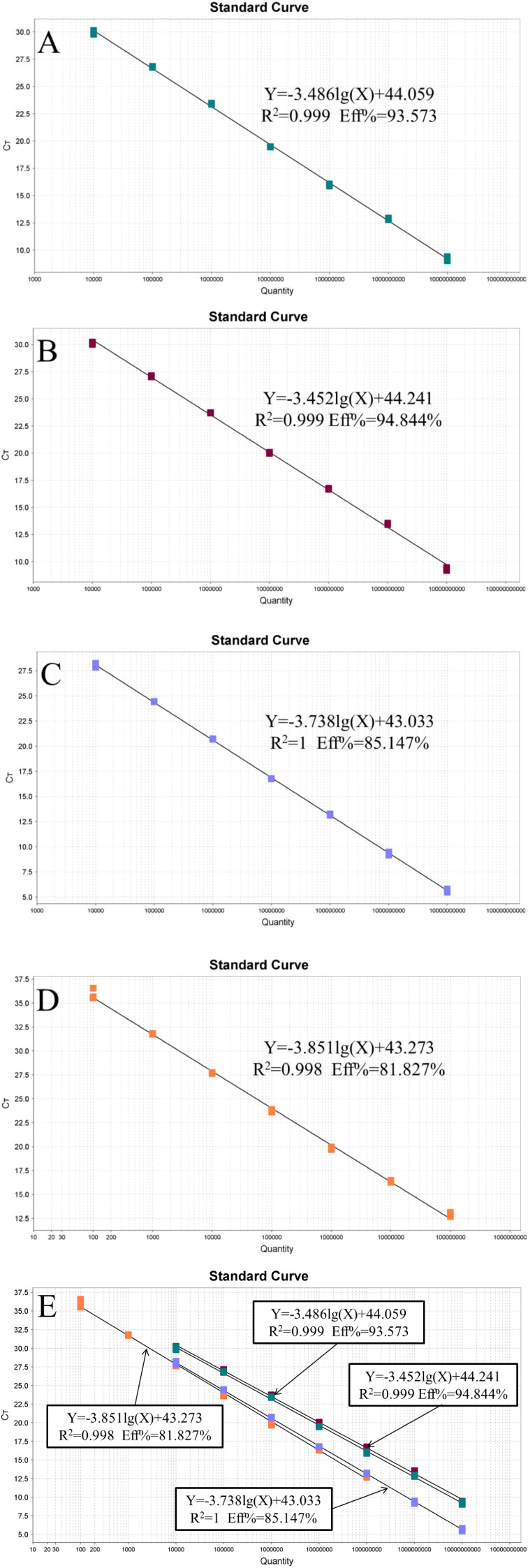
Standard curve of the quadruplex *TaqMan* real-time fluorescence quantitative PCR. **(A)** *Lawsonia intracellularis*; **(B)** *Escherichia coli*; **(C)** *Salmonella*; **(D)** *Brachyspira hyodysenteriae* **(E)** All standard curves.

The recombinant plasmid standards pEASY-invA and pEASY-23s rRNA were prepared at concentrations ranging from 1 × 10^10^ to 1 × 10^4^ copies/μL, while the pEASY-nox standards were prepared from 1 × 10^8^ to 1 × 10^2^ copies/μL to construct standard curves. As shown in **Figure 1**, fluorescence signals were detected in three parallel samples for each concentration gradient. Four standard curves were plotted, with the logarithm of the number of starting templates on the x-axis and the Ct values on the y-axis. The amplification efficiencies were 93.573%, 94.844%, 85.147%, and 81.827%, with corresponding R² values of 0.999, 0.999, 1.000, and 0.998, respectively.

### 3.3 Sensitivity and positive/negative determination results

**Figure 2:**
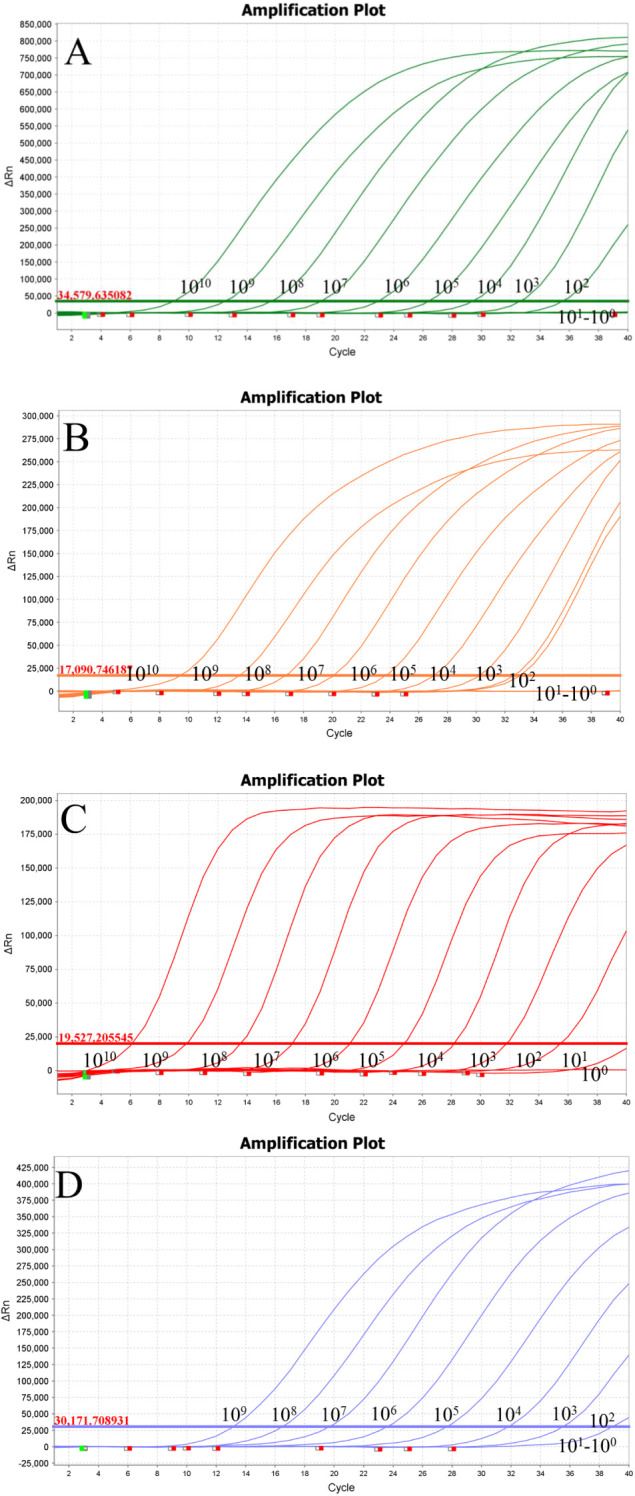
Sensitivity testing. **(A)** Lawsonia intracellularis; **(B)** Escherichia coli; **(C)** Salmonella; **(D)** Brachyspira hyodysenteriae.

The minimum detection concentrations of the established quadruplex TaqMan real-time fluorescence quantitative PCR assay for the pEASY-aspA, pEASY-23s rRNA, and pEASY-nox recombinant plasmid standards were 100 copies/μL, while the lowest detection limit for pEASY-invA was 10 copies/μL (**Figure 2**). Typical S-shaped amplification curves were observed for SE, *E. coli*, LI, and B.h (FAM, VIC, Texas Red, and Cy5), while negative controls (FAM, VIC, Texas Red, and Cy5) showed no amplification curves and Ct values ≥ 40 or no value at all. The test is considered valid if these conditions are met. If the Ct value of the test sample is < 34 and a typical amplification curve is present, it is deemed positive; if 34 ≤ Ct value < 40, the result is classified as suspicious, and the sample should be retested. A Ct value ≥ 40 or no value, along with the absence of a typical amplification curve, indicates a negative result.

### 3.4 Specificity validation results

**Figure 3:**
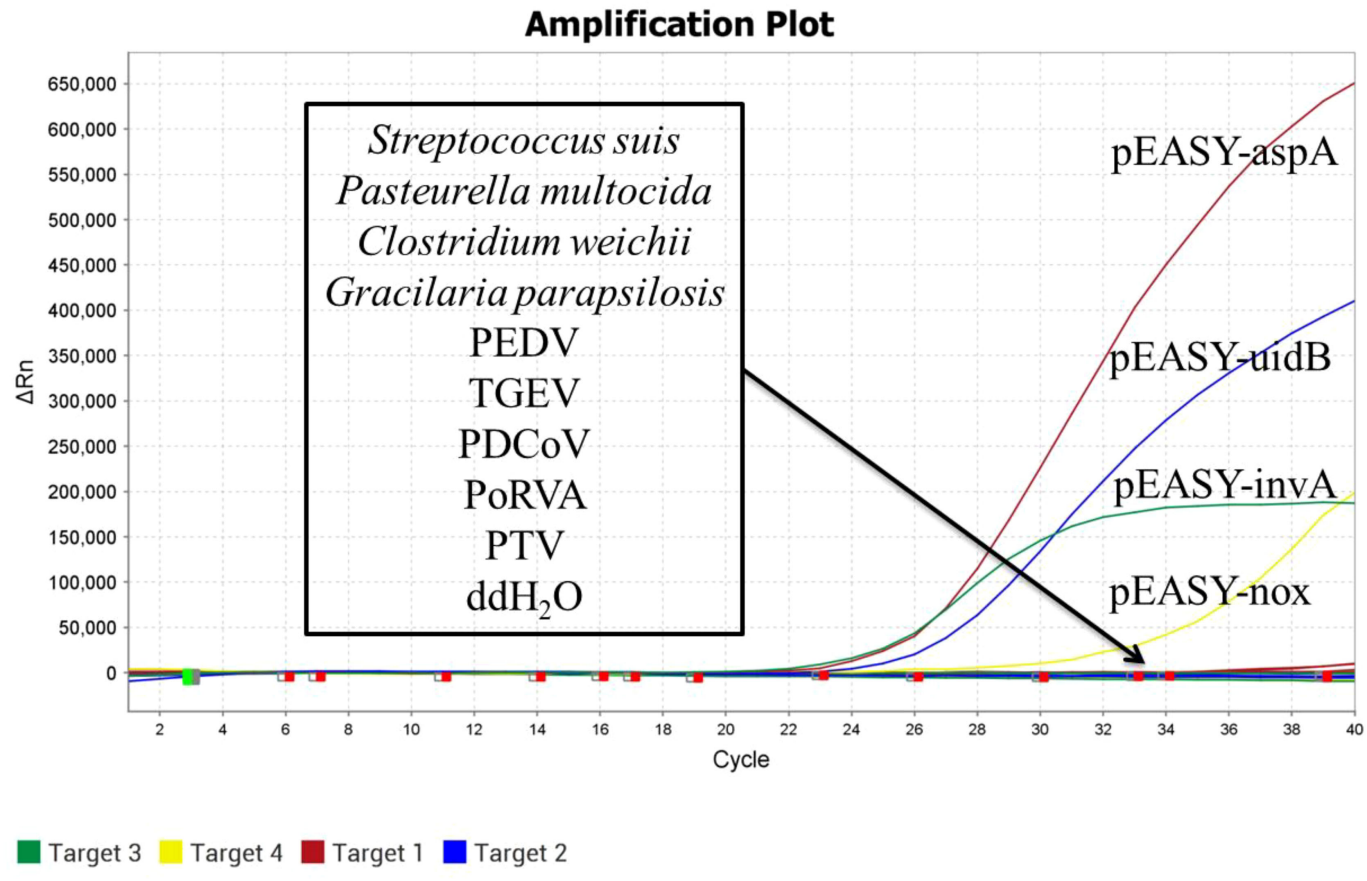
Specificity validation.

DNA/RNA was extracted from SS, PEDV, TGEV, Pm, *Clostridium welchii*, GPS, PDCoV, PoRVA, and PTV as templates. The recombinant plasmid standards pEASY-invA, pEASY-23s rRNA, pEASY-aspA, and pEASY-nox were used as positive controls, with sterile water serving as a blank control. These samples were analyzed using the established quadruplex TaqMan real-time fluorescence quantitative PCR assay. As shown in **Figure 3**, the positive controls produced typical amplification curves, while no amplification curves or Ct values were observed for the nucleic acids extracted from sterile water and non-target pathogens, demonstrating the high specificity of the method.

### 3.5 Anti-interference and repeatability test results

The recombinant plasmids of SE, *E. coli*, LI, and B.h were selected for quadruplex TaqMan real-time fluorescence quantitative PCR amplification at high concentrations of 10^7^ copies/μL and low concentrations of 10^3^ copies/μL. Changes in the thresholds of the amplification curves were observed to assess any potential interference. As shown in **Table 5**, no interference was detected in the amplification of any one high-concentration pathogen against the other three low-concentration pathogens, nor among any three high-concentration pathogens against the low-concentration pathogens. Statistical analysis of the Ct values from the quadruplex TaqMan real-time fluorescence quantitative PCR amplification curves revealed that the coefficients of variation for the Ct values of SE, *E. coli*, LI, and B.h were all less than 1.5% (**Table 6**), indicating good reproducibility in the current experiment.

**Table 5:** Anti-interference test results.

	LI	*E.coli*	SE	B.h
1	10^7^	10^3^	10^3^	10^3^
Ct value	19.66	33.87	31.82	31.70
2	10^3^	10^7^	10^3^	10^3^
Ct value	33.61	20.10	31.08	31.65
3	10^3^	10^3^	10^7^	10^3^
Ct value	33.52	33.99	16.87	31.71
4	10^3^	10^3^	10^3^	10^7^
Ct value	33.01	32.76	31.56	16.316
5	10^3^	10^7^	10^7^	10^7^
Ct value	32.88	19.98	16.64	16.54
6	10^7^	10^3^	10^7^	10^7^
Ct value	19.87	34.01	16.85	16.85
7	10^7^	10^7^	10^3^	10^7^
Ct value	20.01	19.52	31.61	16.34
8	10^7^	10^7^	10^7^	10^3^
Ct value	19.24	19.64	17.01	31.13

**Table 6:** Reproducibility of the quadruplex real time quantitative PCR method.

Standard plasmid	Concentration of template (copies/μL)	Intra-coefficient of variation		Inter-coefficient of variation	
		X ± SD	CV (%)	X ± SD	CV (%)
pEASY-aspA	10^7^	19.657 ± 0.041	0.21	19.234 ± 0.057	0.30
	10^5^	26.629 ± 0.080	0.30	26.610 ± 0.056	0.21
	10^3^	33.601± 0.052	0.15	33.337 ± 0.102	0.31
pEASY-23s rRNA	10^7^	20.077 ± 0.071	0.35	20.150 ± 0.131	0.65
	10^5^	26.981 ± 0.108	0.40	26.864 ± 0.143	0.53
	10^3^	33.885 ± 0.215	0.63	33.645 ± 0.316	0.94
pEASY-invA	10^7^	16.860 ± 0.180	1.07	17.010 ± 0.143	0.84
	10^5^	24.350 ± 0.169	0.69	24.011 ± 0.113	0.47
	10^3^	31.819 ± 0.323	1.01	30.899 ± 0.215	0.70
pEASY-nox	10^7^	16.320 ± 0.183	1.12	16.850 ± 0.103	0.61
	10^5^	21.054 ± 0.100	0.47	24.544 ± 0.213	0.87
	10^3^	31.720 ± 0.138	0.44	31.641 ± 0.350	1.12

### 3.6 Clinical sample results

**Figure 4:**
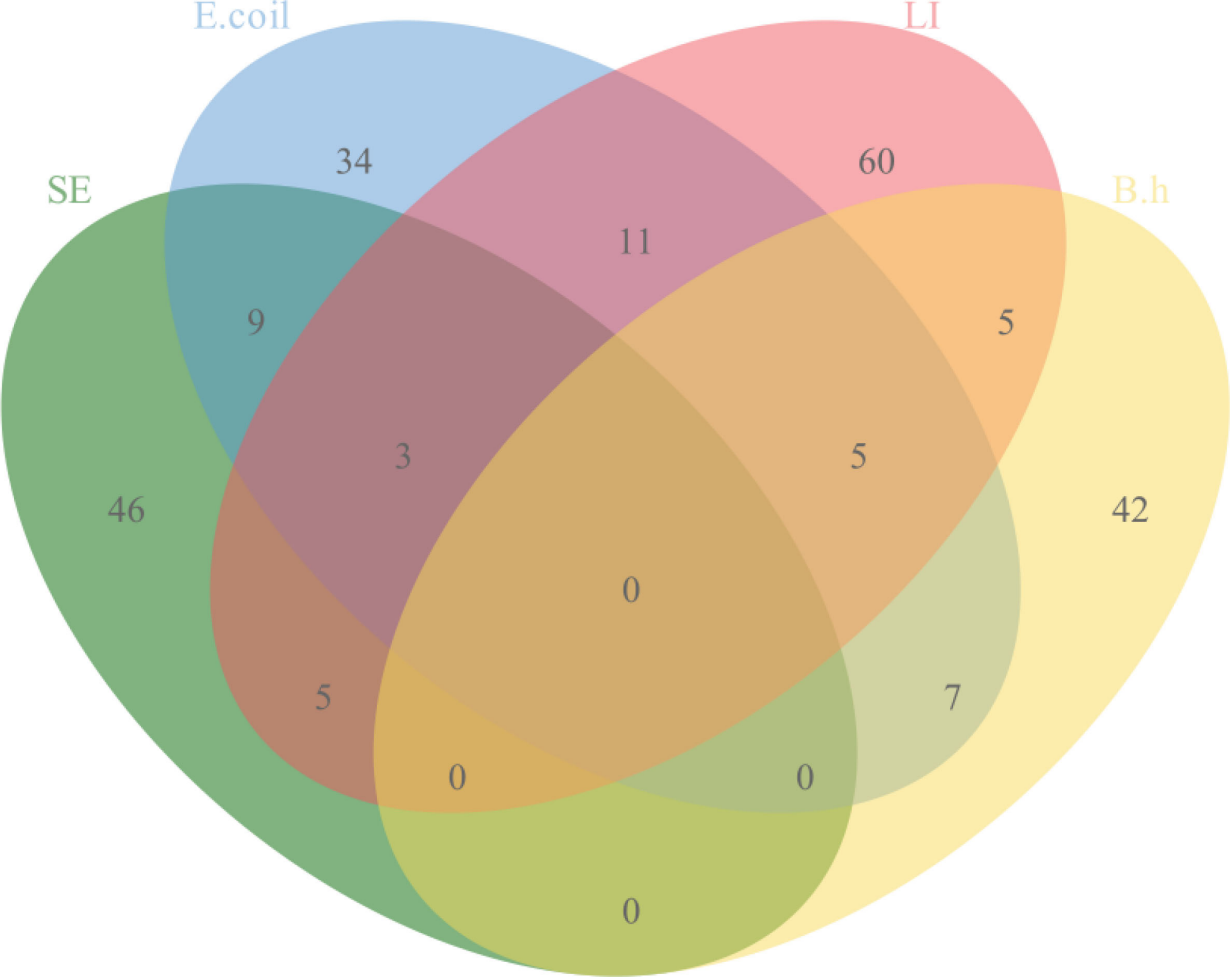
Infection of 227 positive specimens.

The quadruplex TaqMan real-time fluorescent quantitative PCR assay developed in this study, along with the previously reported fluorescent quantitative PCR assay, was used to simultaneously test 263 clinical samples collected. The results indicated that 210 samples were positive and 56 were negative. The positive rates for the pathogens were as follows: SE at 23.95% (63/263), *E. coli* at 26.24% (69/263), LI at 33.84% (89/263), and B.h at 22.43% (59/263). The details of specific mixed infections are presented in **Figure 4**.

## 4 Discussion

Bacterial gastrointestinal diseases pose a significant and prevalent challenge in pig farming, particularly when mixed bacterial infections occur, complicating treatment and leading to substantial economic losses for producers (Arnold et al., 2023; Li L. et al., 2024). For instance, a survey conducted by Zhang et al. found that SE and *E. coli* had a mixed infection rate of 3.64% in pigs (Zhang et al., 2022). Additionally, a study of diarrheal pigs in Germany revealed that 61% of LI and 82.6% of B.h positive herds were affected by mixed infections (Dors et al., 2019). While traditional bacterial isolation and identification methods are considered the “gold standard,” isolating certain bacteria, particularly LI—which is an intracellular parasite with stringent growth requirements—can be extremely challenging in clinical settings (Campillo et al., 2021). Therefore, establishing a rapid, efficient, and simultaneous identification method for these four pathogens is essential for analyzing the epidemiological and pathogenic characteristics of these bacteria, providing a scientific foundation for the development of targeted prevention and control strategies.

In this study, we established a quadruplex TaqMan real-time fluorescent quantitative PCR assay targeting the *invA* gene from SE, the *23S rRNA* gene from *E. coli*, the *aspA* gene from LI, and the *nox* gene from B.h. This assay is both sensitive and specific enough to differentiate between these four pathogens simultaneously. The selection of target genes is crucial for the accuracy of the established method. Given the numerous SE and *E. coli* serotypes that infect pig herds, we chose the *invA* gene, which encodes invasive proteins for SE, and the *23S rRNA* gene for *E. coli* as our targets (Zhang et al., 2022). Both the *invA* and *23S rRNA* genes are species-specific markers for SE and *E. coli*, respectively. Previous molecular biology assays have successfully utilized these genes for detection; for instance, Zhang et al. developed a fluorescent quantitative PCR assay targeting these genes with minimum detection limits of 85.5 copies/μL for SE and 582 copies/μL for *E. coli* (Zhang et al., 2022). In contrast, our method demonstrates enhanced sensitivity, achieving minimum detection limits of 10 copies/μL for SE and 100 copies/μL for *E. coli*.

For LI, traditional antigen detection methods typically employ the *16S rRNA* gene (Wang L. et al., 2024). Wang et al. established a single fluorescent quantitative PCR assay for LI detection, reporting a minimum detection limit of 6.73 × 10^3^ copies/μL (Wang L. et al., 2024). However, the aspartate ammonia lyase gene (*aspA*) of LI is known to be highly conserved (Willems and Reiner, 2010), prompting our selection of this gene as a target; we achieved a minimum detection limit of 100 copies/μL for LI. While *16S rDNA* sequencing has been a common approach for differentiating bacterial species, it has been noted that distinguishing between members of the genus *Brachyspira* based solely on gene sequences can be challenging (Borgström et al., 2017). Therefore, we targeted the NADH oxidase (*nox*) gene of B.h (Rohde et al., 2018), which provided a minimum detection limit of 100 copies/μL.

This study utilized MGB probes in the quadruplex TaqMan real-time fluorescence quantitative PCR assay, which incorporate a 3’-end conjugate that enhances the melting temperature of the probe and stabilizes the probe-target complex. MGB probes are typically shorter than conventional probes, offering improved assay specificity and sensitivity (Li W. et al., 2024). Consequently, the sensitivity of the quadruplex TaqMan real-time fluorescence quantitative PCR assay developed in this study surpasses that of existing fluorescence quantitative PCR assays.

On the other hand, the method established in this study demonstrates high specificity, showing no cross-reactivity with the nucleic acids of nine pathogens, including SS, PEDV, TGEV, Pm, *Clostridium weichii*, GPS, PDCoV, PoRVA, and PTV. The assay exhibits excellent repeatability, with coefficients of variation both between and within batches being less than 1.5%. Furthermore, the four templates do not interfere with one another, regardless of whether they are present at high or low concentrations.

Clinical sample testing revealed positive rates of 23.95% for SE, 26.24% for *E. coli*, 33.84% for LI, and 22.43% for B.h. Notably, the occurrence of double, triple, and quadruple infections suggests that single infections are no longer the primary source of economic loss in the swine industry; rather, mixed infections involving two or more pathogens represent the main epidemiological trend (Ito et al., 2020). The 36 diarrheal samples in which target pathogens were not detected may have been attributed to non-infectious factors or other pathogens, such as PEDV and TGEV (Du et al., 2021; Chen et al., 2024; Garcias et al., 2024). Therefore, it is essential for clinical practices to consider a range of factors comprehensively and implement effective measures to prevent and control related diseases.

## 5 Conclusion

In summary, this study has developed a sensitive, specific, rapid, and efficient quadruplex TaqMan real-time fluorescence quantitative PCR assay for the simultaneous identification of *Salmonella enterica*, *Escherichia coli*, *Lawsonella intracellularis*, and *Brachyspira hyodysenteriae*. The goal is to provide technical support for the development of effective measures aimed at preventing and treating gastrointestinal diseases in pigs.

## Data Availability

The original contributions presented in the study are included in the article/supplementary material. Further inquiries can be directed to the corresponding authors.
